# Fracture heuristics: surgical decision for approaches to distal radius fractures. A surgeon’s perspective

**DOI:** 10.3205/iprs000110

**Published:** 2017-05-22

**Authors:** Florian Wichlas, Serafim Tsitsilonis, Sebastian Kopf, Björn Dirk Krapohl, Sebastian Manegold

**Affiliations:** 1 Center for Musculoskeletal Surgery, Charité - University Medicine Berlin, Berlin, Germany; 2 Department for Plastic Surgery and Hand Surgery, St. Marien Hospital, Berlin, Germany

**Keywords:** distal radius fracture, heuristics, surgical approach, fracture classification

## Abstract

**Introduction:** The aim of the present study is to develop a heuristic that could replace the surgeon’s analysis for the decision on the operative approach of distal radius fractures based on simple fracture characteristics.

**Patients and methods:** Five hundred distal radius fractures operated between 2011 and 2014 were analyzed for the surgeon’s decision on the approach used. The 500 distal radius fractures were treated with open reduction and internal fixation through palmar, dorsal, and dorsopalmar approaches with 2.4 mm locking plates or underwent percutaneous fixation. The parameters that should replace the surgeon’s analysis were the fractured palmar cortex, and the frontal and the sagittal split of the articular surface of the distal radius.

**Results:** The palmar approach was used for 422 (84.4%) fractures, the dorsal approach for 39 (7.8%), and the combined dorsopalmar approach for 30 (6.0%). Nine (1.8%) fractures were treated percutaneously. The correlation between the fractured palmar cortex and the used palmar approach was moderate (r=0.464; p<0.0001). The correlation between the frontal split and the dorsal approach, including the dorsopalmar approach, was strong (r=0.715; p<0.0001). The sagittal split had only a weak correlation for the dorsal and dorsopalmar approach (r=0.300; p<0.0001).

**Discussion:** The study shows that the surgical decision on the preferred approach is dictated through two simple factors, even in the case of complex fractures.

**Conclusion:** When the palmar cortex is displaced in distal radius fractures, a palmar approach should be used. When there is a displaced frontal split of the articular surface, a dorsal approach should be used. When both are present, a dorsopalmar approach should be used. These two simple parameters could replace the surgeon’s analysis for the surgical approach.

## Zusammenfassung

**Einleitung:** Das Ziel der vorliegenden Arbeit ist es, eine heuristische Methode zu entwickeln, die es ermöglicht anstelle einer aufwendigen Frakturanalyse eine Wahl des Operationszugangs zur Versorgung der distalen Radiusfraktur basierend auf einfachen Charakteristika zu treffen.

**Patienten und Methoden:** Fünfhundert distale Radiusfrakturen, die zwischen 2011 bis 2014 operiert wurden, wurden hinsichtlich der chirurgischen Zugangswahl untersucht. Radiusfrakturen wurden entweder über einen palmaren, dorsalen oder einen kombinierten dorsopalmaren Zugang offen reponiert und mittels 2,4 mm winkelstabiler Plattenosteosynthese versorgt, oder unterzogen sich nach geschlossener Reposition einer perkutanen Osteosynthesetechnik. Die Frakturcharakteristika, die die präoperative Analyse des Chirurgen bei der Zugangswahl ersetzen sollen, waren: frakturierte palmare Kortikalis sowie koronare und sagittale Frakturlinie in der Gelenkfläche.

**Ergebnisse:** Der palmare Zugang wurde bei 422 (84,4%) Frakturen, der dorsale Zugang bei 39 (7,8%) und ein kombinierter dorsopalmarer Zugang bei 30 Frakturen (6,0%) verwendet. Neun (1,8%) Frakturen wurden geschlossen reponiert und perkutan stabilisiert. Die Korrelation zwischen „frakturierte palmare Kortikalis“ und „palmarer Zugang“ war moderat: (r=0,464; p<0,0001). Die Korrelation zwischen „frontaler Split in der Gelenkfläche“ und „dorsaler Zugang inkl. dorsopalmarer Zugang“ war hoch: (r=0,715; p<0,0001). Der Parameter „sagittaler Split“ zeigte nur eine schwache Korrelation mit dem „dorsopalmaren Zugang“ (r=0,300; p<0.0001).

**Diskussion:** Die Studie zeigt, dass die Wahl des chirurgischen Zugangs selbst bei der Versorgung von komplexen distalen Radiusfrakturen durch zwei einfache Parameter bestimmt wird.

**Schlussfolgerung:** Wenn die palmare Kortikalis frakturiert ist, sollte ein palmarer Zugang gewählt werden. Wenn eine dislozierte Frakturlinie in der Koronarebene verläuft, dann sollte der dorsale Zugang angewendet werden. Wenn beide Parameter vorliegen, sollte der kombinierte dorsopalmare Zugang angewendet werden. Die Verwendung dieser beiden einfachen Parameter ersetzt die aufwendige Frakturanalyse bei der Wahl des Operationszugangs.

## Introduction

Heuristics permit to reduce complex analysis to simple decision making with a few factors. Their property to be “fast and frugal eliminates most cognitive bias” [1]. Especially in complex fractures, experienced surgeons analyze sophistically the fracture pattern to decide upon the surgical approach and the reduction sequence. They use their experience to choose the best surgical approach for distal radius fractures. Their goal for surgical treatment is the most anatomic fracture reduction possible. 

To achieve this goal, locking compression plates were designed that fit the anatomy of the distal radius. They can be positioned through palmar and dorsal approaches. Both permit sufficient reduction and provide good functional results [2], [3]. Approach-related complication rates, such as tendon ruptures or complex regional pain syndrome seemed to be similar for both approaches in recent studies [4], [5]. 

Preoperative analyses of distal radius fractures include radiographical examination with X-rays and in complex fractures computer tomography (CT) scans [6]. Comminuted fractures need a meticulous preoperative evaluation in order to choose the most suitable surgical approach that enables the best reduction sequence and result.

When operating on distal radius fractures, certain reduction limitations exist: the displaced palmar cortex can be controlled and reduced directly only through a palmar but not through a dorsal approach. The anatomic reduction of displaced frontal splits of the articular surface can be controlled only through dorsal arthrotomy. On the other hand, displaced sagittal splits can be directly reduced and controlled using both approaches, and the palmar tilt can be controlled either indirectly through a palmar or directly through a dorsal approach. The usage of an image intensifier during surgery is very helpful to control fracture reduction; however, especially in detecting intraarticular steps in distal radius fractures it has certain limitations that are inherent to the system [7]. These facts imply that the displaced palmar cortex and the displaced frontal split of the articular surface would dictate the surgical approach of distal radius fractures. 

Unfortunately, most of the existing classifications for distal radius fractures fail to have an impact on surgical decision-making, especially in complex fractures [8]. Thus, the decision is based on the experience and the “gut feeling” of the surgeon; that is where heuristics come into play, as they “refer to experience-based techniques for problem solving” [9]. 

The aim of this study was to identify surgical decision patterns over the preferred surgical approach of distal radius fractures and to identify a heuristic for systematic decision-making regarding the most appropriate surgical approach based on simple fracture characteristics. 

## Patients and methods

**Figure 1:**
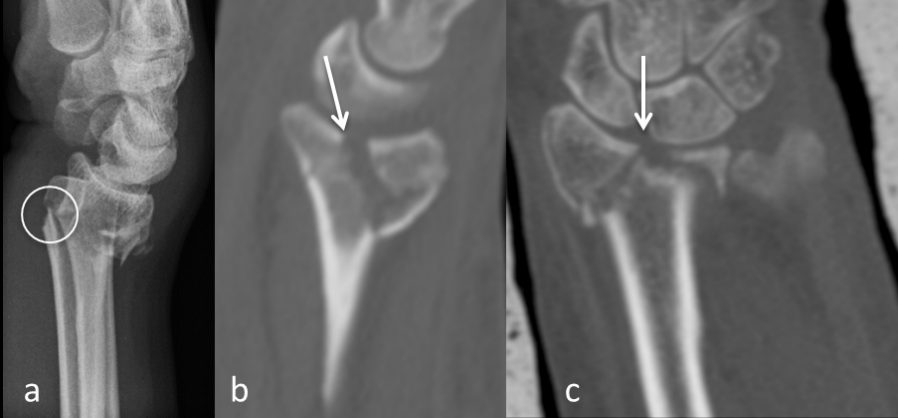
a: The parameter displaced palmar cortex in a conventional sagittal radiograph (circle). b: The parameter displaced frontal split in a sagittal plane of a CT-scan (arrow). c: The parameter displaced sagittal split in a frontal plane of a CT-scan (arrow). The main parameters of the heuristic.

We retrospectively analyzed 500 consecutive distal radius fractures (476 patients) that were operated in our university hospital (level 1 trauma center) between 2011 and 2014. Patient data were acquired through our hospital databank. Demographic data, surgical reports, radiographs and CT scans were evaluated. Radiographs were analyzed pre- and postoperative in two planes. CT scans were evaluated in case of a radiologically identified intraarticular fracture. Major anatomic characteristics of the operated fractures were thoroughly analyzed based on the preoperative radiologic diagnostics in search of similarities and differences that lead to the surgical decision. The suspected main parameters evaluated on their impact on the decision of the surgical approach and for the development of the heuristic in these fractures were:

- The displaced palmar cortex (Figure 1a (Fig. 1)).
- The displaced frontal split of the articular surface extending dorsally in the metaphysis (Figure 1b (Fig. 1)).
- The displaced sagittal split of the articular surface (Figure 1c (Fig. 1)).

Undisplaced fracture parameters were not considered.

Firstly, we searched for correlations between the parameters and the surgical approach. Then we investigated how often these parameters were present in the different approach groups. Finally, we examined which approach was used when the parameter was present or not.

Moreover, the following parameters were investigated for correlations:

- AO/OTA classification
- The classic instability criteria [9]:
  - Intraarticular fracture
  - Dorsal comminution zone
  - Dorsal tilt >20°

The surgical reports were scanned for the approach and the used implant.

The surgical technique was described in detail previously [10]. An abridged version of the technique is provided in the present article. The palmar approach was centered over the flexor carpi radialis tendon between the radial artery and the flexor tendons through the pronator quadratus muscle. The dorsal approach was centered over Lister’s tubercle and leads through the third extensor sheath to the dorsal aspect of the distal radius. The fourth extensor sheet was elevated subperiostally to reach the dorsoulnar aspect of the radius. The radial styloid was accessed between the first and the second extensor sheet.

The decision on the surgical approach was made the day before surgery including a drawn planning of the fracture, the reduction and the implants. Afterwards it was discussed in the “preoperative meeting”, where all surgeries of the upcoming day are discussed with all available colleagues of our department. Surgery was performed or assisted by consultants specialized in orthopedic trauma surgery. Whenever an intraoperative change of the strategy was necessary concerning the approach, the consultant made that decision. 

Continuous variables were expressed as means ± standard deviation (SD), whereas categorical variables were documented as percentages [%]. The Kolmogorov-Smirnov test was used in order to check distribution normality. For parametric variables the Student t-test was used for the comparison of two groups; for non-parametric variables the Mann-Whitney test was implemented. Differences for categorical variables were assessed with the Fisher’s exact test. Correlations were examined with either Pearson’s or the Spearman’s rank correlation coefficient depending on the distribution of analyzed data. Differences were considered statistically significant if the null hypothesis could be rejected with >95% confidence (p<0.05). 

## Results

**Figure 2:**
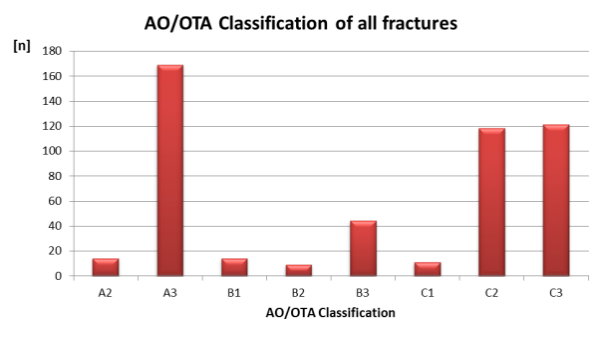
AO/OTA classification of all fractures

The majority of patients were women (289 women and 187 men) with a mean age of 57.1 years (standard deviation (SD) 18.6 years, range 16–95 years) in our collective. In 289 cases the left side was fractured, in 211 the right, and in 24 both sides. The results of the AO/OTA classification are provided in Figure 2 (Fig. 2).

Locking compression plates (2.4 mm; DePuy-Synthes^®^, Umkirch, Switzerland) were implanted in 491 fractures. Nine patients with an isolated fracture of the radial styloid process (AO/OTA type B1) were treated percutaneously with one 3.5 mm cortical lag screw.

The palmar approach was used for 422 (84.4%) fractures, the dorsal approach for 39 (7.8%), and the combined dorsopalmar approach for 30 (6.0%). Nine (1.8%) fractures were treated percutaneously.

The distributions of the used approaches in relation to our main fracture parameters (displaced palmar cortex, displaced frontal split and displaced sagittal split) are provided in Table 1 (Tab. 1), Table 2 (Tab. 2), Table 3 (Tab. 3), and Table 4 (Tab. 4). The correlation between the displaced palmar cortex and the palmar approach was moderate [(r=0.464 (p<0.0001)]. Fractures operated through the palmar approach had a displaced palmar cortex in 95.0% and no frontal split in 92.2%. 

The correlation between the frontal split and the dorsal approach, including the dorsopalmar approach, was strong [r=0.715 (p<0.0001)]. Fractures operated through the dorsal approach had a displaced frontal split in 74.4% and no displaced palmar cortex in 79.5%. 

The correlation between the sagittal split and the dorsal approach, including the dorsopalmar approach, was weak [r=0.300 (p<0.0001)]. Fractures operated through the dorsal approach had a displaced sagittal split in 53.8% and those operated from palmar 27.5%. 

The parameters “palmar cortex” and/or “no frontal split” had led the surgeons to decide for a palmar approach. The parameter “frontal split” led to a dorsal and dorsopalmar approach, and the parameter “no palmar cortex” to a dorsal approach.

**Figure 3:**
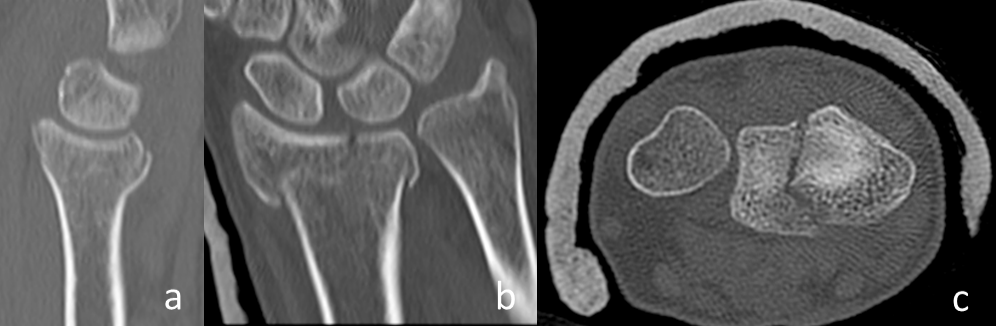
a: CT in sagittal plane showing the palmar cortex is not displaced. b: CT in frontal pane showing a sagittal split of the articular surface. c: CT in axial plane showing a sagittal split of the articular surface. Distal radius fracture (AO type C2) with a non-displaced palmar cortex (i.e. no palmar cortex) and no frontal split.

The fact that only displaced fracture parameters have been taken in account for the decision of the surgical approach led to a subgroup of fractures that had paradoxically no palmar cortex and no frontal split as these fracture parameters were not displaced (Figure 3 (Fig. 3)). These fractures did not follow the before mentioned observation. In these cases, the palmar cortex was fractured very far distally and the articular surface was impacted in the metaphysis. We observed 31 of these fractures in our collective: 7 were operated from dorsal, 16 from palmar, and 8 were fractures of the radial styloid process (type B1) that were treated percutaneously. 

### AO/OTA classification 

**Figure 4:**
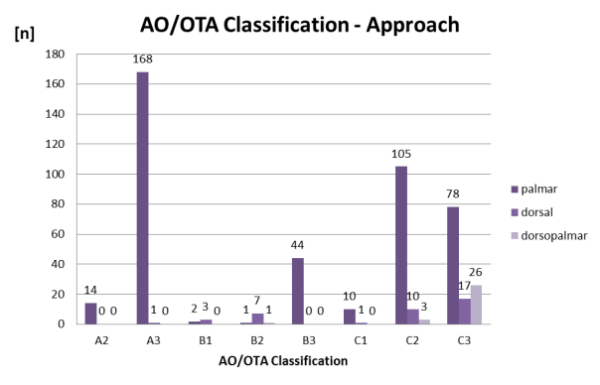
Incidence of AO/OTA classified fractures in the approach groups

Type A2, A3, B3, and C1 fractures were mainly operated from palmar and B2 fractures from dorsal (Figure 4 (Fig. 4)). Among all distal radius fractures, the dorsal and dorsopalmar approach was predominantly used in C2 and C3 type fractures (56 of 239 C2/C3 fractures; 23.4%). However, most of these C2 and C3 type fractures were operated through a palmar approach (n=183 of 239 C2/C3 fractures; 76.6%). Nevertheless, there was a weak correlation between increasing severity of the fractures (from A2 to C3) and a more frequent use of the dorsal and dorsopalmar approach [r=0.345 (p<0.0001)].

Regarding instability criteria, the correlation between intraarticular fractures and the dorsal and dorsopalmar approach was weak [r=0.278 (p<0.0001)]. There was no correlation between the approaches and the dorsal comminution zone [r=0.028 (p=0.537)] or the dorsal tilt >20° [r=0.069 (p=0.127)].

## Discussion

As Marewski stated, “heuristics are simple decision strategies that ignore part of the available information, basing decisions on only a few relevant predictors” [11]. In contrast to complex algorithms that try to cover every single possibility, heuristics ask simple “yes-or-no” questions. They are shortcuts that reduce complex analysis to simplify the process of decision-making. They are based on human experiences and knowledge and extract the relevant factors for decision-making [12]. Exceptions to that heuristic would still need the surgeon’s own analysis and decision. 

The present heuristic is based on the idea that anatomical reduction is the goal for articular fractures. As already mentioned, the absolute anatomic reduction of fracture lines and splits can only be controlled directly to be sure that desired result has been achieved. Image intensifier monitoring cannot detect sufficiently every step or gap; though direct vision and fracture control should be the goal. While anatomic reduction might not be necessary for every aspect of the distal radius (e.g. for the dorsally comminuted zone), the articular surface, radial length, and palmar tilt should be restored [13], [14], [15]. Referring to our results, a fractured palmar cortex and the presence of a frontal split seem to represent the main pathology that lead the surgeons to the choice of the surgical approach. 

The heuristic has to address two simple questions in decision-making over the surgical approach of complex distal fractures: Is the palmar cortex displaced? And, is there a displaced frontal split? 

When the palmar cortex is displaced, a palmar approach should be used. When the frontal split is displaced, a dorsal approach should be used. When the frontal split was not displaced or absent, a palmar approach should be used. When the palmar cortex and frontal split are displaced, a dorsopalmar approach should be used. These two simple questions seemed to be sufficient to replace the surgeon’s analysis for the surgical approach in our collective.

Attempts to simplify treatment options for distal radius fractures have been made before. Many classifications exist to categorize distal radius fractures regarding clinical outcome, fracture morphology, preoperative displacement, and fracture stability [16]. Most of them have a poor inter- and intra-observer reliability and seem to lack surgical relevance [16], [17]. For example, the commonly used AO/OTA classification was developed to reflect the severity of the fracture and therefore predict its clinical outcome. Unfortunately, many studies showed that these goals were achieved with limited success [18], [19]. The AO/OTA classification recommends dorsal approaches mainly for (complex) C-type fractures. In the present study, most of the C fractures were operated through a palmar approach. Up to now, there are no specific or proved guidelines that recommend a dorsal or dorsopalmar approach for distal radius fractures. A simple strategy for operative treatment options regarding the surgical approach of fractures instead of classifications would be of importance. 

Regarding the morbidity, the literature does not provide a clear recommendation for a certain approach, because there is no decisive advantage of one approach over the other [20], [21]. The choice can be made by the surgeon based on the notion of what can be reduced through which approach. The fracture pathology dictates the best possible approach. As most fractures are typical Colles fractures (A2–3 and C-type fractures) with an extension mechanism, the most common approach used is the palmar approach, as was the case in our collective too. This approach is simple, fast, safe and does not require high surgical expertise. The surgical reduction sequence for these fractures is similar for most cases: first, anatomic reduction of the palmar cortex and then restoration of the palmar tilt. In these fractures the palmar cortex is involved with or without a sagittal split and they are mostly addressed through a palmar approach. 

In the present analysis, the sagittal split had no major impact on the decision which approach to choose. A possible explanation for that could be that these splits can be both, reduced from palmar and dorsal. A palmar approach permits the anatomic reduction on the palmar side of the split, although without intra-articular control. The dorsal approach permits direct intra-articular access to the split through arthrotomy. The instability criteria showed some statistic significant results. That may be referred to the fact that these criteria are interrelated with each other. A dorsal tilt >20° is usually combined with a dorsally comminuted fracture zone. Most of these criteria reflect the mechanism of extension fractures. However, their use in the daily routine for the choice of the correct surgical approach is limited. The main reason is that they were defined as parameters for stability assessment and not for the surgical approach. Moreover, their assessment is sometimes difficult when it comes to determining when they are present or not. A fracture of the ulnar styloid, for instance, is not always easy to diagnose, even when a CT scan is performed. Degenerative pathologies of the triangular fibrocartilage complex (TFCC) make it sometimes impossible to distinct between ossifications and fractures. The diagnosis of a dorsally comminuted zone on X-ray films is difficult in many cases, too. It is obvious that fractures treated from dorsal or dorsopalmar are more often intra-articular fractures because they are the ones that would require an arthrotomy to control the articular surface.

Nevertheless, there were exceptions too. In some radial styloid fractures, e.g. type B1, the heuristic did not seem to apply. They represent a special subgroup as their pathology can be often adequately addressed with closed reduction and percutaneous screw fixation. When the styloid fragment is big enough to require plate fixation, the heuristic can be used because a displaced palmar cortex would indicate a palmar approach, while the lack of it would favor a dorsal one. Certainly, associated pathologies like scapholunar dissociations influence the choice of the approach.

The interesting group of “neither palmar cortex nor frontal split fractures” need further evaluation (Figure 3 (Fig. 3)). These fractures are usually not grossly displaced and often amendable to conservative treatment. Their indication for surgery is mainly a dorsal tilt greater than 20 degrees. In these cases, both approaches are feasible: a palmar approach where the tilt is restored with reduction over the plate; a dorsal approach with direct reduction of the impaction. Indirect reduction in these fractures is usually not possible because the joint is impacted in the metaphysis. 

**Figure 5:**
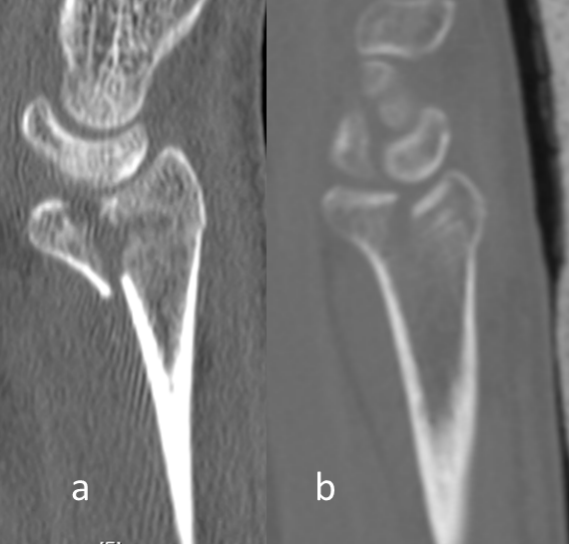
a: CT in sagittal plane of a distal radius fracture with a palmar extending frontal split. b: CT in sagittal plane of a distal radius fracture with a dorsal extending frontal split. Difference between frontal splits.

As mentioned above, the frontal split needs to be defined as “extending dorsally in the metaphysis”, because when it extends in the palmar cortex it is an AO type B3 fracture that typically requires a palmar approach (Figure 5 (Fig. 5)). The displaced palmar extending frontal split (AO type B3) is the exact opposite pathology of a displaced dorsally extending frontal split (AO type B2) that requires a dorsal approach. 

We emphasize, that the two parameters for the heuristic need to be displaced in order to be taken into account. The reason for this is that undisplaced fracture lines usually have no surgical consequences. Certainly, there are fracture lines where the decision of being displaced or not, is difficult. We were considering defining cut-offs of gaps and steps, but they would not have any rational base. A reasonable definition would be the one of a displacement that does not require reduction. Another possibility would be to define displacement as fracture lines that cannot be controlled indirectly.

Of course, other factors have to be taken into account besides concomitant lesions. In our experience, a dorsopalmar approach takes much longer in the operating room and leads to a significant increased postoperative swelling of the hand than the other approaches. This leads to a longer hospital stay in our institution. The preoperative soft tissues must be critically evaluated. Some osteoporotic multi-fragmentary fractures in aged patients may not need perfect reduction of “destroyed” joints, rather than a restoration of length and tilt to form a secondary articular surface, as the clinical outcome is not necessarily inferior [22]. 

A prospective randomized controlled trial is required to confirm whether this heuristic offers clinically relevant advantages.

## Conclusions

In decision-making upon distal radius fractures, the displaced palmar cortex und frontal split seem to be crucial.

## Notes

### Competing interests

The authors declare that they have no competing interests.

## Figures and Tables

**Table 1:**
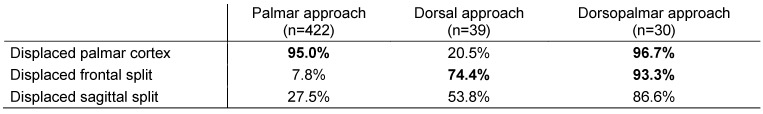
This table shows how often the parameter (displaced palmar cortex, displaced frontal split, or displaced sagittal split) was present when an approach (palmar, dorsal, or dorsopalmar) was used. The prevalence is expressed in percentages. Nine fractures were treated percutaneously.

**Table 2:**
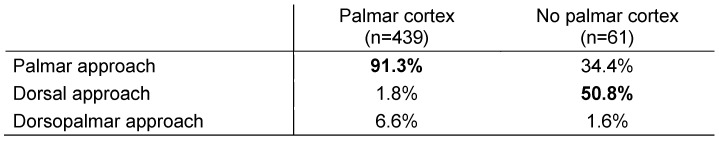
The table shows how often an approach (palmar, dorsal, or dorsopalmar) was used when the parameter “palmar cortex” was present. The prevalence is expressed in percentages. Nine fractures were treated percutaneously.

**Table 3:**
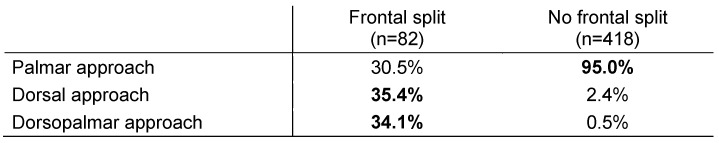
The table shows how often an approach (palmar, dorsal, or dorsopalmar) was used when the parameter “frontal split” was present. The prevalence is expressed in percentages. Nine fractures were treated percutaneously.

**Table 4:**
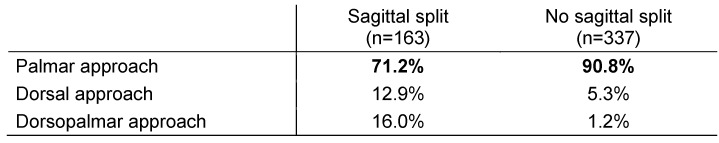
The table shows how often an approach (palmar, dorsal, or dorsopalmar) was used when the parameter “sagittal split” was present. The prevalence is expressed in percentages. Nine fractures were treated percutaneously.

## References

[1] Gigerenzer G, Goldstein DG (1996). Reasoning the fast and frugal way: models of bounded rationality. Psychol Rev.

[2] Chou YC, Chen AC, Chen CY, Hsu YH, Wu CC (2011). Dorsal and volar 2.4-mm titanium locking plate fixation for AO type C3 dorsally comminuted distal radius fractures. J Hand Surg Am.

[3] Matschke S, Wentzensen A, Ring D, Marent-Huber M, Audigé L, Jupiter JB (2011). Comparison of angle stable plate fixation approaches for distal radius fractures. Injury.

[4] Yu YR, Makhni MC, Tabrizi S, Rozental TD, Mundanthanam G, Day CS (2011). Complications of low-profile dorsal versus volar locking plates in the distal radius: a comparative study. J Hand Surg Am.

[5] Wichlas F, Haas NP, Disch A, Machó D, Tsitsilonis S (2014). Complication rates and reduction potential of palmar versus dorsal locking plate osteosynthesis for the treatment of distal radius fractures. J Orthop Traumatol.

[6] Medoff RJ (2005). Essential radiographic evaluation for distal radius fractures. Hand Clin.

[7] Dahlen HC, Franck WM, Sabauri G, Amlang M, Zwipp H (2004). Fehlklassifikation extraartikulärer distaler Radiusfrakturen in konventionellen Röntgenaufnahmen. Vergleichende Untersuchung der Frakturmorphologie zwischen biplanarer Röntgendiagnostik und CT. Unfallchirurg.

[8] Hunt JJ, Lumsdaine W, Attia J, Balogh ZJ (2013). AO type-C distal radius fractures: the influence of computed tomography on surgeon's decision-making. ANZ J Surg.

[9] Cooney WP, Linscheid RL, Dobyns JH (1979). External pin fixation for unstable Colles' fractures. J Bone Joint Surg Am.

[10] Jupiter JB, Ring DC (2004). AO Manual of Fracture Management: Hand and Wrist.

[11] Marewski JN, Gigerenzer G (2012). Heuristic decision making in medicine. Dialogues Clin Neurosci.

[12] Gigerenzer G, Todd PM, ABC Research Group (1999). Simple Heuristics That Make Us Smart.

[13] Gruber G, Gruber K, Giessauf C, Clar H, Zacherl M, Fuerst F, Bernhardt GA (2008). Volar plate fixation of AO type C2 and C3 distal radius fractures, a single-center study of 55 patients. J Orthop Trauma.

[14] Trumble TE, Schmitt SR, Vedder NB (1994). Factors affecting functional outcome of displaced intra-articular distal radius fractures. J Hand Surg Am.

[15] Ring D, Prommersberger K, Jupiter JB (2004). Combined dorsal and volar plate fixation of complex fractures of the distal part of the radius. J Bone Joint Surg Am.

[16] Jupiter JB, Fernandez DL (1997). Comparative classification for fractures of the distal end of the radius. J Hand Surg Am.

[17] Kural C, Sungur I, Kaya I, Ugras A, Ertürk A, Cetinus E (2010). Evaluation of the reliability of classification systems used for distal radius fractures. Orthopedics.

[18] Bolmers A, Luiten WE, Doornberg JN, Brouwer KM, Goslings JC, Ring D, Kloen P (2013). A comparison of the long-term outcome of partial articular (AO Type B) and complete articular (AO Type C) distal radius fractures. J Hand Surg Am.

[19] Sonderegger J, Schindele S, Rau M, Gruenert JG (2010). Palmar multidirectional fixed-angle plate fixation in distal radius fractures: do intraarticular fractures have a worse outcome than extraarticular fractures? Arch Orthop Trauma Surg. http://dx.doi.org/10.1007/s00402-010-1045-z.

[20] Rein S, Schikore H, Schneiders W, Amlang M, Zwipp H (2007). Results of dorsal or volar plate fixation of AO type C3 distal radius fractures: a retrospective study. J Hand Surg Am.

[21] Rausch S, Schlonski O, Klos K, Gras F, Gueorguiev B, Hofmann GO, Mückley T (2013). Volar versus dorsal latest-generation variable-angle locking plates for the fixation of AO type 23C 2.1 distal radius fractures: a biomechanical study in cadavers. Injury.

[22] Arora R, Gabl M, Gschwentner M, Deml C, Krappinger D, Lutz M (2009). A comparative study of clinical and radiologic outcomes of unstable colles type distal radius fractures in patients older than 70 years: nonoperative treatment versus volar locking plating. J Orthop Trauma.

